# Ligand-to-Metal Ratio Governs Radical-Scavenging Ability of Malate-Stabilised Ceria Nanoparticles

**DOI:** 10.3390/nano14231908

**Published:** 2024-11-27

**Authors:** Arina D. Filippova, Alexander E. Baranchikov, Maria A. Teplonogova, Irina V. Savintseva, Anton L. Popov, Vladimir K. Ivanov

**Affiliations:** 1 Kurnakov Institute of General and Inorganic Chemistry, Russian Academy of Sciences, Leninsky Prospect, 31, Moscow 119991, Russia; 2 Institute of Theoretical and Experimental Biophysics, Russian Academy of Sciences, Institutskaya Str., 3, Pushchino 142290, Russia

**Keywords:** ceria, sols, surface, grafting density, alkyl peroxyl radicals

## Abstract

Cerium dioxide sols stabilised with L-malic acid were shown to exhibit significant antioxidant activity towards alkyl peroxyl radicals in the range of ligand:CeO_2_ molar ratios of 0.2–1 (0.2:1, 0.4:1, 0.5:1, 0.6:1, 0.8:1 and 1:1). The antioxidant activity of cerium dioxide nanoparticles greatly depended on L-malic acid content and increased by 8 times when the ligand:CeO_2_ molar ratio increased from 0.2:1 to 0.4:1. An estimate of the ligand:CeO_2_ molar ratio required to ensure complete surface coverage of CeO_2_ nanoparticles with malate anions resulted in a value of 0.2. Aggregation degree of CeO_2_ nanoparticles depends on the ligand:CeO_2_ molar ratio. In the range of ligand:CeO_2_ molar ratios 0.2–0.4, the size of aggregates decreased by an order of magnitude. The antioxidant capacity of 1 mM malate-stabilised cerium dioxide (0.2:1) relative to sodium ascorbate was 0.012 ± 0.001 mM. The antioxidant activity of cerium dioxide stabilised with L-malic acid at a ligand:CeO_2_ molar ratio of 0.2:1 was 80 times less than the antioxidant activity of sodium ascorbate. Cerium dioxide nanoparticles stabilised with L-malic acid did not demonstrate a cytotoxic effect against human mesenchymal stem cells, in a wide range of concentrations (10^−3^–10^−5^ M), and their proliferation was stimulated after 72 h of cultivation. The results obtained show new possibilities for the design of biocompatible ceria-based nanomaterials with tunable pro- and antioxidant properties; these materials can further be assessed in view of their potential for treating oxidative stress-related disorders.

## 1. Introduction

The disruption of redox homeostasis and the development of oxidative stress are considered to be among the causes of neurodegenerative [1,2,3], cardiovascular [4,5,6] and autoimmune [7,8] diseases. In this regard, the pharmacological action of many promising therapeutic drugs is based on maintaining the pro-/antioxidant balance in living organisms [9,10,11,12]. In particular, fine regulation of redox status in cellular organelles is possible with the use of inorganic nanomaterials (nanozymes) that exhibit activities characteristic of natural antioxidant enzymes and compounds with radical-scavenging activity [13]. Importantly, the research unveiling the enzyme-like behaviour of nanomaterials (nanozymes) was among the Clarivate’s Top 10 Research Fronts in chemistry and materials science [14], as well the IUPAC Top Ten Emerging Technologies in Chemistry [15]. This behaviour is governed by the specifics of the interaction of nanomaterials with various radical species (including, for example, reactive oxygen species, such as peroxyl radicals) and by the balance of their anti- and pro-oxidant abilities.

Currently, ceria-based materials are considered the most promising among nanozymes, due to their high biocompatibility [16,17], selective cytotoxicity [18] and recyclable pro- and antioxidant activity [19,20,21,22], including enzyme-like activity [23,24]. The bioactivity of nanocrystalline cerium dioxide enables its application as a component of antibacterial and anti-inflammatory preparations [25,26,27], as well as in compositions for the regeneration of wounds of various origins [28,29,30].

In therapeutic applications of nanomaterials, it is convenient to use drugs in the form of colloidal solutions to provide the precise dosage of the active substance. The long-term stability of CeO_2_ sols is usually achieved through their modification with biocompatible ligands (citrate, dextran, polyethylene glycol [31,32,33,34]), but in order to develop new biomedical preparations, it is necessary to evaluate the possibility of using other stabilisers. It is important to note that the content of the ligand on the surface of nanoparticles is known to have a significant impact on the stability of colloidal solutions and the physicochemical properties of colloidal particles. For example, Tombácz et al. showed that the range of pH stability of magnetite sol expands with increasing grafting density of Fe_3_O_4_ nanoparticles with citric and gallic acid [35]. Zhang et al. found that the lattice constant increases with an increase in the degree of coverage of cerium dioxide nanoparticles with oleic acid [36]. Of great importance is the surface charge that cerium dioxide particles acquire as a result of interaction with ligands. Collin et al. showed that CeO_2_ nanoparticles with a positive surface charge are significantly more toxic to *C. elegans* nematodes than those that are negatively charged or neutral [37].

Importantly, the surface modification of metal oxide nanoparticles, including CeO_2_, can occur spontaneously during their interaction with the components of biological milieu: proteins and polybasic acids [38], amino acids [39] and inorganic oxyanions [40,41,42]. A good candidate for stabilising colloidal solutions of oxide nanoparticles is malic acid, which participates in the tricarboxylic acid cycle (Krebs cycle) and has its own antioxidant and cardioprotective activities [43]. Derivatives of malic acid, for example, polymalic acid (PMLA), can be used to create drug delivery carriers [44,45,46].

A quantitative assessment of the antioxidant activity of inorganic nanomaterials can be performed using a chemiluminescent method [47,48]. Methods for analysing antioxidant activity based on the use of chemiluminescent probes (luminol, lucigenin, coumarin, etc.) provide high sensitivity and selectivity for the determination of various types of free radicals. Chemiluminescent methods for studying radical-scavenging properties make it possible to express the activity of artificial antioxidants in units of antioxidants such as ascorbic acid, trolox or α-tocopherol [49].

In this paper, for the first time, an in-depth study of the antioxidant activity of cerium dioxide sols stabilised with L-malic acid with different ligand:CeO_2_ molar ratios (from 0.1:1 to 1:1) is presented. The study of radical-scavenging activity was carried out using a chemiluminescent method based on the interaction of luminol with 2,2′-azobis(2-amidinopropane) dihydrochloride acting as a source of alkyl peroxyl radicals. An analysis of the antioxidant activity of cerium dioxide sols enabled an evaluation of the possibility of its precise regulation by varying the ligand-to-metal ratio for ceria nanoparticles and L-malic acid. The antioxidant capacity of malate-stabilised colloidal solutions of cerium dioxide relative to the antioxidant sodium ascorbate was analysed. Finally, an analysis of the cytotoxicity of malate-stabilised cerium dioxide sols was carried out using the MTT assay and Live/Dead assay on a culture of human mesenchymal stem cells.

## 2. Materials and Methods

### 2.1. Materials

The following chemicals were used as starting compounds: ammonium cerium(IV) nitrate (99.9%, Lanhit, Moscow, Russia), anhydrous L-malic acid (≥99%, Sigma-Aldrich, St. Louis, MO, USA), aqueous ammonia solution (99.9%, Chimmed, Moscow, Russia), isopropyl alcohol (99.9%, Chimmed), 2,2′-azobis(2-amidinopropane) dihydrochloride (440914, Sigma-Aldrich), luminol (123072, Sigma-Aldrich), tris-hydrochloride (10812846001, Sigma-Aldrich), sodium ascorbate (≥99%, Sigma-Aldrich) and Milli-Ω Water (18.2 MΩ/cm, Merck Millipore, St. Louis, MO, USA).

### 2.2. Synthesis of CeO_2_ Sols

Ceric ammonium nitrate (2.33 g) was dissolved in 23 mL of deionised water. The resulting solution was placed in a 100 mL Synthware^TM^ (Beijing Synthware Glass Inc., Beijing, China) autoclave (25% filling degree) and heated at 95 °C for 24 h. The resulting yellow precipitate was separated by centrifugation (20,000 rpm, 5 min), washed three times with isopropyl alcohol, re-dispersed in 25 mL of deionised water and boiled for 2 h to remove the remaining isopropanol. The concentration of cerium dioxide in the sol obtained was 25.3 g/L (0.147 M). The resulting ceria sol (pH 2.0) was stabilised with L-malic acid in the range of ligand:CeO_2_ molar ratios of 0.1:1–1:1. In this case, highly opalescent colloidal solutions were formed, which became transparent after the pH was adjusted to 7.4–7.6 with 3 M aqueous ammonia solution. Before the analysis of pro-/antioxidant activity, the resulting CeO_2_ sols were diluted to the same concentration, c = 20.7 g/L (0.12 M).

### 2.3. Methods of Characterisation

The sample sols were first dried at 40 °C. Powder X-ray diffraction analysis (XRD) of the dry sols was performed using a Bruker (Billerica, MA, USA) D8 Advance diffractometer (CuKα radiation), in the angular range of 20–90°2 Θ, in steps of 0.02°2 Θ and with a signal acquisition time of 0.4 s per step. Full-profile analysis of XRD patterns was performed using TOPAS v.4.2 software (Billerica, MA, USA) and diffraction maxima were fitted to Voigt pseudo-functions.

The optical absorption spectra of the sols obtained were recorded in a 200–900 nm range, at 0.1 nm steps, on an SF-2000 spectrophotometer (OKB Spectr, Saint-Petersburg, Russia) with a deuterium-halogen light source.

CeO_2_ nanoparticles were investigated using a Tescan Amber GMH (Tescan Group, Brno-Kohoutovice, Czech Republic) scanning electron microscope (SEM) at an accelerating voltage of 30 kV, using an R-STEM detector. A drop of the aqueous sol was placed on a formvar/carbon Cu grid (Ted Pella Inc., Redding, CA, USA) and left to evaporate. The acquisition of images was performed in dark field mode at ×300k magnification.

The IR spectra of the samples were recorded in attenuated total reflection geometry, using an InfraLUM FT-08 IR spectrometer Spectrum 65 (Lumex, Saint-Petersburg, Russia) with a spectral resolution of 1 cm^−1^, in the wavenumber range of 400–4000 cm^−1^.

The Raman spectra were recorded using a Confotec NR500 spectrometer (SOL Instruments, Minsk, Belarus) with a 514 nm laser, using a 20× objective (NA = 0.45) at ~2 mW laser power. The spectral resolution was 2.1 cm^−1^.

Dynamic light scattering (DLS) and ζ-potential measurements were carried out at 20 °C, using a Photocor Compact-Z analyser (Photocor, Moscow, Russia) equipped with a 636.65 nm laser. The correlation function for each sample was obtained by averaging ten curves, each being acquired for 20 s. The hydrodynamic diameter of aggregates was determined using DynalS software (Moscow, Russia).

Pro-/antioxidant activity of CeO_2_ sols was investigated using the chemiluminescent method, with a reaction of luminol oxidation in the presence of alkyl peroxyl radicals in a Tris-HCl (100 mM, pH 7.4) buffer solution at 36 °C. The thermolabile compound 2,2′-azobis(2-amidinopropane) dihydrochloride (ABAP) was used as a source of alkyl peroxyl radicals. The background luminescence was recorded for 2 min after the addition of a mixture of ABAP (47.8 μM) and luminol (5 mM) in a buffer solution. Then, an aliquot (4.6–18.4 μL) of CeO_2_ sol was added (0.55–2.2 mM) and a chemiluminescent signal was recorded for 60 min. As the controls, the solutions containing Tris-HCl buffer, ABAP and luminol were used, and no nanoparticles were added. The values of antioxidant or pro-oxidant capacity were calculated by numerical integration of the inhibition area in the chemiluminescent curves over time, using PowerGraph software v.3.3 (Moscow, Russia).

A study of the cytotoxicity of CeO_2_ nanoparticles stabilised in ligand:CeO_2_ molar ratios of 0.5:1 and 1:1 was carried out on a culture of primary human mesenchymal stem cells derived from the third molar bud, which were extracted from a healthy 16-year-old patient by orthodontic indications. The cultivation was carried out in DMEM/F12 medium (1:1) with the addition of 10% fetal bovine serum (FBS) and 100 U/mL penicillin/streptomycin and 2 mM L-glutamine, at 37 °C and in an atmosphere of 5% CO_2_. The introduction, seeding and cultivation of samples, to enable a study of the level of dehydrogenase activity and assess the cytotoxicity and viability of cells (staining with Hoechst 33342 and propidium iodide), were carried out in a similar way. The seeding was carried out in 96-well plates in DMEM/F12 medium (1:1), with the addition of 10% fetal bovine serum (FBS), 100 U/mL penicillin/streptomycin and 2 mM L-glutamine. The cell seeding density was 30,000/cm^2^. Cultivation was carried out at 37 °C in an atmosphere of 5% CO_2_. After 10 h of cultivation, various concentrations of CeO_2_ nanoparticles were added to the vials. Wells with cells without the addition of CeO_2_ nanoparticles were used as a control.

An analysis of the metabolic activity of cells after cultivation with CeO_2_ nanoparticles in the concentration range of 10^−3^–10^−5^ M was carried out using the MTT assay. This is a colourimetric test for assessing the metabolic activity of cells, based on the reduction of tetrazolium dye 3-(4,5-dimethylthiazol-2-yl)-2,5-diphenyl-tetrazolium bromide into insoluble formazan by NADPH-dependent oxidoreductase enzymes. At least five wells in a plate were taken for each experimental point. The MTT test was carried out after 24, 48 and 72 h of cultivation with nanoparticles. The optical density of the formazan formed was determined using an INNO-S plate reader (LTec. Co., Seoul, Republic of Korea), at a wavelength of 540 nm. The optical density values obtained are presented as a percentage of the untreated control.

The viability of cells was assessed by staining with fluorescent dyes Hoechst 33,342 and propidium iodide, 24, 48 and 72 h after the addition of CeO_2_ nanoparticles. Intercalating dye propidium iodide (λ_ex_ 546 nm, λ_em_ 575−640 nm) penetrates only into dead cells and stains their nuclei. The fluorescent dye Hoechst 33342 (λ_ex_ 355 nm, λ_em_ 460 nm) stains the DNA of all cells in the culture, which makes it possible to quantify the percentage of dead cells from the total number of cells in the culture. Micrographs of cell cultures were obtained using a ZOE imager (BioRad, Hercules, CA, USA), followed by quantitative assessment using the ImageJ program v. 1.54i.

The data are presented as mean ± standard deviation, unless otherwise stated. Statistical analysis was carried out using the two-tailed Student’s *t*-test. Statistical analysis was performed using the GraphPrism Program, version 8.0.1 (Dotmatics, Boston, MA, USA).

## 3. Results

**Figure 1:**
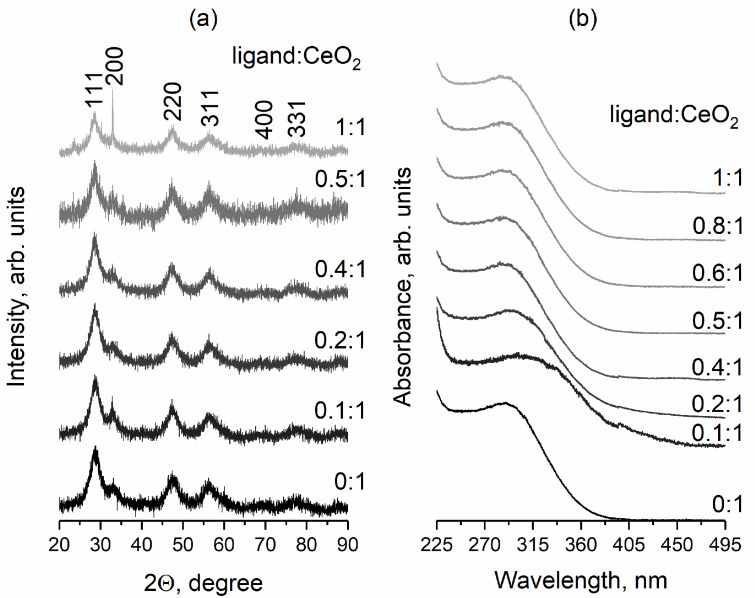
XRD patterns of CeO_2_ powders obtained by drying ceria sols stabilised with L-malic acid (**a**) and UV–vis absorption spectra of cerium dioxide sols (**b**). Molar ratios ligand:CeO_2_ are shown in the Figure.

The thermohydrolysis of ceric ammonium nitrate at 95 °C led to the formation of a stable colloidal solution of cerium dioxide whose aggregates were about 15 nm in size [50]. According to XRD data, the sol obtained contained nanocrystalline cerium dioxide (space group Fm$\bar{3}$m, PDF2 034-0394) with a crystallite size in the range of 2.8−3.7 nm (Figure 1a).

According to UV–vis absorption spectroscopy data (Figure 1b), all CeO_2_ sols obtained presented an absorption band in the wavelength range of 280–300 nm, corresponding to an absorption band of nanoscale cerium dioxide with a bandgap energy of ~3.4 eV, which agrees well with the literature data [51]. For the malate-stabilised ceria sol obtained at the ligand:CeO_2_ molar ratio of 0.1:1, a red shift at the absorption band edge was observed that was probably caused by Rayleigh scattering.

In the STEM image (Figure S1a) of non-stabilised cerium dioxide sol, one can observe CeO_2_ particles with a diameter of less than 10 nm. The stabilisation of ceria sol with L-malic acid at ligand:CeO_2_ molar ratios of 0.2:1, 0.4:1 and 1:1 did not have a significant effect on the morphology and degree of ceria nanoparticle aggregation (Figure S1b–d).

**Figure 2:**
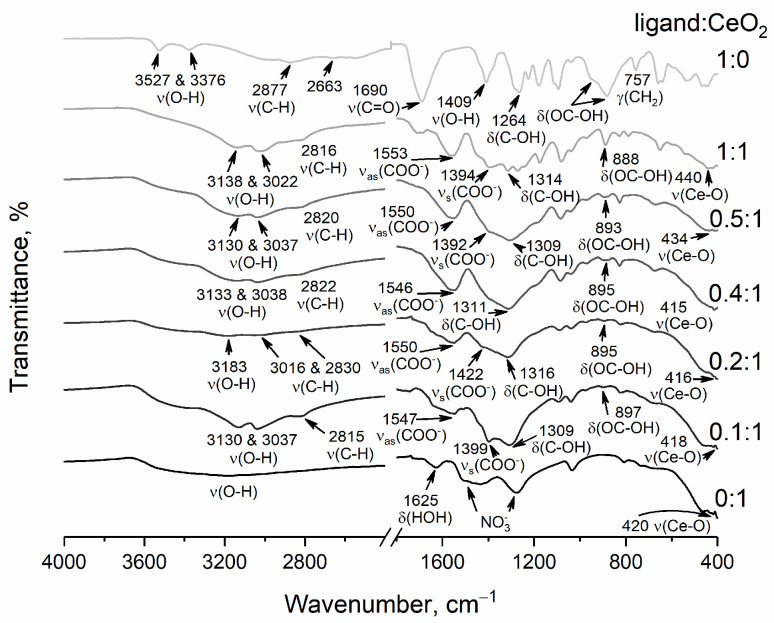
FTIR spectra of the powders obtained by drying ceria sols stabilised with L-malic acid. Molar ratios ligand:CeO_2_ are shown in the Figure.

The IR spectrum of non-stabilised cerium dioxide sol (Figure 2) shows a broad absorption band in the 3550–3200 cm^−1^ region (antisymmetric and symmetric υ(O-H) vibrations) and in the ranges of 1630–1600 cm^−1^ (δ(HOH)), 1530–1480 cm^−1^, 1290–1250 cm^−1^ (vibrations of nitrate species adsorbed on CeO_2_ surface) and 440–420 cm^−1^ (valence vibrations of Ce-O) [52]. L-malic acid (Figure 2) is characterised by absorption bands at 3527 and 3376 cm^−1^ (υ(O-H)), at 2877 cm^−1^ (υ(C-H)), 1690 cm^−1^ (υ(C=O) carboxyl groups), 1409 cm^−1^ (υ(O-H)) and 1264 cm^−1^ (δ(C-OH)), and in the range of 960–880 cm^−1^ (δ(C(O)OH) carboxyl groups) [53].

IR spectra (Figure 2) of malate-stabilised CeO_2_ sols show absorption bands in the ranges of 3200–2500 cm^−1^ (υ(O-H) of the bound hydroxyl group) and of 3000–2840 cm^−1^ (υ(C–H)). The formation of carboxylate complexes on the surface of cerium dioxide particles is confirmed by the presence of absorption bands of the carboxyl group in the range of 1610–1550 cm^−1^ (asymmetric υ(COO^−^) vibrations) and in the range of 1450–1400 cm^−1^ (symmetric υ(COO-) vibrations), whereas the absorption band at 1690 cm^−1^ (υ(C=O)) disappears [54,55,56,57]. In the IR spectra (Figure 2), a shift of the band of the hydroxyl group (δ(C-OH)) from 1264 to 1316 cm^−1^ is an observed characteristic of the oxyacid chelate complexes [52].

Similar absorption bands are observed in the IR spectrum of ammonium malate (see Figure S2) in the range of 1610–1550 cm^−1^ (asymmetric υ(COO^−^) vibrations), in the range of 1450–1400 cm^−1^ (symmetric υ(COO^−^) vibrations) and in the region of 1720–1670 cm^−1^ (υ(C=O)), characteristic of monosubstituted salts of malic acid [58,59]. In the IR spectrum of individual malic acid, the absorption band corresponding to asymmetric υ(COO^−^) vibrations is absent, whereas the IR spectra of both ammonium malate and malate-stabilised CeO_2_ sols show this band [53]. In contrast to L-malic acid, in the spectra of ammonium malate and malate-stabilised ceria, the C=O deformation vibrations absorption band is absent (758 cm^−1^ for L-malic acid), while the band corresponding to carboxyl scissor vibrations σ(COO^−^) is present (see Figure S2) [60]. In the IR spectra of malate-stabilised ceria, the position of the latter band (830 cm^−1^) is notably shifted relative to its position in the spectrum of ammonium malate (790 cm^−1^).

**Figure 3:**
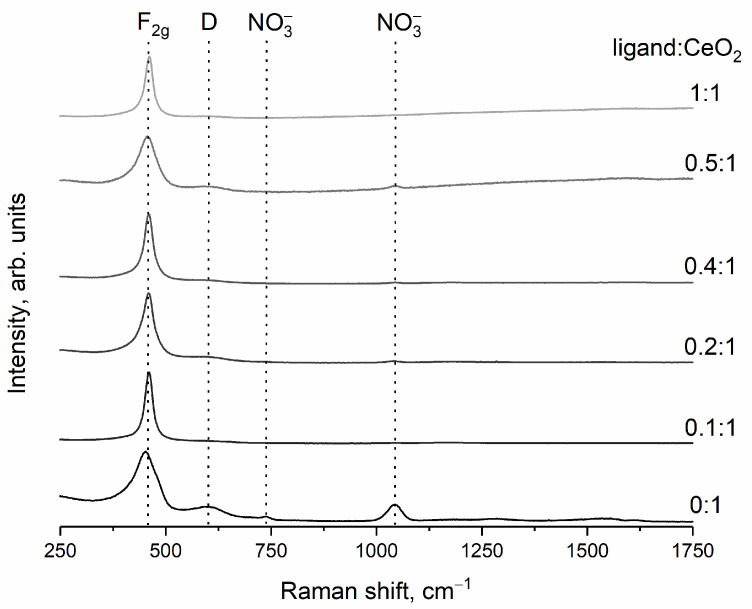
Raman spectra of CeO_2_ powders obtained by drying ceria sols stabilised with L-malic acid. Molar ratios ligand:CeO_2_ are shown in the Figure.

Raman spectra of dried ceria sols (Figure 3) show an intense band in the 450–460 cm^−1^ region, which can be attributed to the F_2g_ mode (symmetric stretching mode of the Ce−O_8_ vibrational unit) [61,62]. A weak band of about 600 cm^−1^ can be ascribed to the defect-induced (D) mode [63,64]. The bands at 736 and 1044 cm^−1^ are characteristic of the bending and stretching vibrations of nitrate species adsorbed on the oxide surface [65].

**Figure 4:**
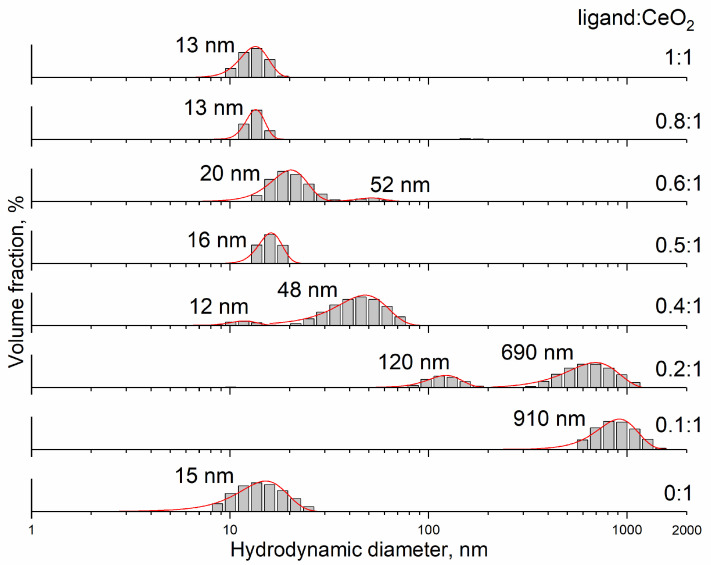
Distributions of hydrodynamic diameters in aqueous ceria sols stabilised with L-malic acid with 0:1−1:1 ligand:CeO_2_ molar ratios. The pH value of the non-stabilised sol was about 2. The pH value of malate-stabilised sols was approximately 7.4.

According to dynamic light scattering data, non-stabilised cerium dioxide sol (at pH 2.0) is characterised by a monomodal size distribution of aggregates with a mean hydrodynamic diameter of 15 nm (Figure 4). When CeO_2_ sol (pH 2.0) was added dropwise to an aqueous solution of L-malic acid (pH 1.5–2.2), highly opalescent (almost opaque), light yellow colloidal solutions were formed (pH ~1.3) with a CeO_2_ aggregate size of more than 100 nm. This confirms the sorption of negatively charged malate ions on the positively charged ceria surface resulting in the decrease in ζ-potential of the nanoparticles (see Table S1). Note that the mixed sol containing both CeO_2_ nanoparticles and malic acid has lower pH than its individual components (bare CeO_2_ sol and malic acid solutions), which also confirms the chemical interaction between malate ions and the ceria surface. In order to obtain the cerium dioxide sols stabilised by L-malic acid with high colloidal stability, the pH of the mixtures was adjusted to 7.4 with an aqueous ammonia. Such pH-adjusted CeO_2_@L-malic acid sols at low ligand:CeO_2_ molar ratios (0.1:1 and 0.2:1) retained a tendency for particle aggregation (Figure 4), which is in line with the low absolute values of ζ-potential (–7 mV and –13 mV, respectively) of the nanoparticles (see Table S1). This can be due to the incomplete coverage of the surface of the nanoparticles with malate ions. Conversely, colloidal solutions stabilised by the malate anion at ligand:CeO_2_ molar ratios of 0.4:1–1:1 were transparent, indicating good colloidal stability of the sols. The ζ-potential values for these sols amounted to –15…–17 mV (see Table S1) supporting the supposition that, for these sols, the ceria surface was completely covered by malate ions. From Figure 4, it can be seen that, with an increase in the ligand:CeO_2_ molar ratio from 0.1:1 to 0.5:1, the size of aggregates in CeO_2_ colloids decreased from 910 nm to 16 nm. For the sols with the ligand:CeO_2_ molar ratios of 0.5:1–1:1, the degree of aggregation differed insignificantly and the average aggregate size was ~16 nm. Note that a sharp change in the degree of particle aggregation occurred in the range of the ligand:CeO_2_ molar ratios of 0.2–0.4.

The ζ-potential value of non-stabilised CeO_2_ sol (pH 2.0) was positive, 18 ± 1 mV. The ζ-potential values of malate-stabilised cerium dioxide sols were in the range of −7 to −17 mV, which further confirmed the formation of malate complexes on the surface of cerium dioxide particles and the stabilisation of colloidal solutions.

It is most likely that the aggregation degree of cerium dioxide sols depends on the degree of surface coating of ceria nanoparticles with L-malic acid. The ligand:CeO_2_ molar ratio required to ensure complete coverage of CeO_2_ nanoparticles was assessed from geometric considerations in the rigid sphere approximation (Formula 1, Table 1). It should be taken into account that this assessment is approximate, and the actual area of a ligand footprint is at least half of its topological polar surface area (TPSA). The ligand:CeO_2_ ratio of 0.18:1 is consistent with the above dynamic light scattering results (Figure 4).

(1) $$ligand:{CeO}_{2}=\frac{C_{ligand}}{C_{{CeO}_{2}}}=\frac{N_{ligand}}{N_{A}\cdot C_{{CeO}_{2}}}=\frac{S_{particle}\cdot N_{particle}}{S_{ligand}\cdot N_{A}\cdot C_{{CeO}_{2}}}\;\;\;\;\;\;\;\;\;\;\;\;\;\;\;\;\;\;\;\;\;\;\;\;\;\;\;\;\;\;\;\;\;\;\;=\frac{\pi \cdot D_{XRD}^{2}\cdot C_{{CeO}_{2}}\cdot a^{3}\cdot N_{A}\cdot 6}{(\frac{TPSA}{2})\cdot N_{A}\cdot C_{{CeO}_{2}}\cdot 4\cdot \pi \cdot D_{XRD}^{3}}=\frac{3{\cdot a}^{3}}{TPSA\cdot D_{XRD}}$$

The antioxidant activity of malate-stabilised ceria sols towards alkyl peroxyl radicals in a neutral medium was investigated. The source of free radicals, 2,2′-azobis(2-amidinopropane) dihydrochloride, decomposes at 36 °C to form alkyl radicals R·, which interact with oxygen to form alkyl peroxyl radicals ROO. The radical-scavenging properties of citrate-stabilised ceria sols were studied previously using a similar method [67].

At the very beginning of the measurements, a fast and reversible quenching of a chemiluminescence signal occurs due to the addition of the material under study. This effect takes a very short time (~1 min) and virtually does not affect the values of antioxidant or pro-oxidant activity (capacity), which are calculated by integrating the inhibition area in the chemiluminescence curves over time.

A chemiluminescence signal on a longer scale can show quite a complex behaviour. During the interaction of antioxidants (radical-scavengers) with alkyl peroxyl radicals in the presence of luminol, a decrease in a chemiluminescence signal is observed, and the signal approaches zero. The specific shape of a chemiluminescence curve depends strongly on the values of reaction rate constants associated with the interaction with reactive species [68]. In some cases, the chemiluminescence signal can increase over control, indicating the pro-oxidant behaviour of a material. Importantly, the pro-oxidant behaviour of a material does not mean that it is an oxidiser. This pro-oxidant behaviour merely indicates that the material does not scavenge reactive species, but enhances (e.g., catalyses) the formation of radicals from radical source molecules (e.g., ABAP).

In the presence of strong antioxidants, the complete suppression of a chemiluminescence signal is followed by a rapid return of a signal intensity to baseline, indicating high rate constants for their reactions with radicals. The area of the region of luminescence suppression (the difference between the kinetic curve for an antioxidant and a control) is proportional to the quantity of radical species scavenged by an antioxidant [69,70]. Obviously, this effect is directly proportional to the concentration of an antioxidant in a reaction medium.

**Figure 5:**
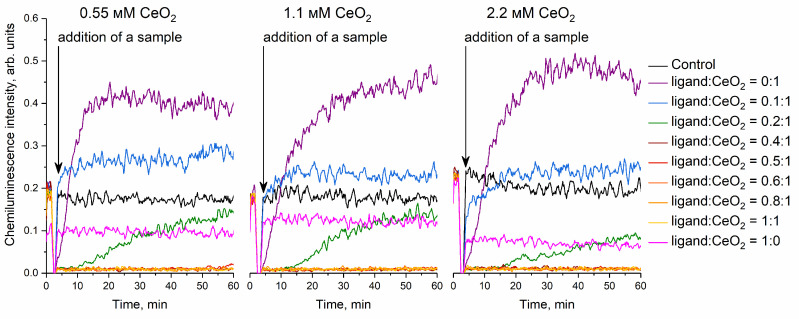
Kinetic curves chemiluminescence for the luminol oxidation product in a reaction mixture containing a Tris-HCl buffer solution (pH 7.4), ABAP and cerium dioxide sols stabilised with L-malic acid at molar ratios ligand:CeO_2_ 0:1−1:1. The concentration of cerium dioxide in the reaction mixture is shown in the Figure. The chemiluminescence signal was recorded at 36 °C. As the control, the solution containing Tris-HCl, ABAP and luminol was used, without the addition of ceria nanoparticles.

The addition of non-stabilised CeO_2_ sol (ligand:CeO_2_ molar ratio 0:1) to the reaction mixture containing a Tris-HCl buffer solution (pH 7.4), luminol and 2,2′-azobis(2-amidinopropane) dihydrochloride led to an increase in the intensity of the chemiluminescence of the product oxidation of luminol (Figure 5) compared with the control solution, indicating the pro-oxidant activity of cerium dioxide towards alkyl peroxyl radicals. Previously, Datta et al. showed that pristine cerium dioxide (particle size ~40 nm) exhibited pro-oxidant activity in the human colorectal carcinoma cell line [71].

Solutions containing only L-malic acid (0.55, 1.1 and 2.2 mM) were characterised by the suppression of chemiluminescence intensity compared with the control solution (Figure 5). A decrease in the glow intensity of the oxidation product of luminol in the presence of malic acid allows it to be classified as an antioxidant. It is known that hydroxy acids present radical-scavenging activity and maintain the redox balance in living systems [72,73]. In addition, Wu et al. showed that L-malic acid reduces the accumulation of reactive oxygen species and reduces the level of lipid peroxidation in the liver of aged rats by increasing the activity of antioxidant enzymes (superoxide dismutase and glutathione peroxidase) [74]. Lu at el. demonstrated that malic acid inhibits the spontaneous combustion of coal by reducing the concentration of free radicals by up to 2-fold [75].

The stabilisation of the CeO_2_ sol with L-malic acid at a ligand:CeO_2_ molar ratio of 0.1:1 led to a decrease in the pro-oxidant activity of cerium dioxide by up to 2 times (Figure 5). As can be seen from Figure 5, cerium dioxide sol stabilised with L-malic acid at a ligand:CeO_2_ molar ratio of 0.2:1 exhibited antioxidant activity against alkyl peroxyl radicals. A similar type of chemiluminescence kinetic curve is characteristic of the antioxidant trolox [76]. Increasing the ligand:CeO_2_ molar ratio to 0.4:1–1:1 led to a significant increase in the antioxidant activity of malate-stabilised CeO_2_ sols (Figure 5). It is probable that the kinetics of free radical-scavenging and luminol oxidation in the presence of malate-stabilised ceria sols is determined by the ligand adsorption–desorption equilibrium on the surface of cerium dioxide nanoparticles. Note that the radical-scavenging activity of L-malic acid itself (at a concentration comparable with the concentration of malic acid in the reaction mixture containing a CeO_2_ sol stabilised at a ligand:CeO_2_ molar ratio of 1:1) is lower than the activity of cerium dioxide sols stabilised by L-malic acid at a ligand:CeO_2_ molar ratio of 0.4−1.

Previously, Pota et al. demonstrated that modification of cerium dioxide nanoparticles (5–20 nm in diameter) with melanin more than doubles the radical-scavenging activity of CeO_2_ against reactive oxygen and nitrogen species in human dermal fibroblast cells [77]. Similarly, Hu et al. showed that, with increasing grafting density of catechol-substituted polyethylene glycol on the surface of ceria nanoparticles, their antioxidant activity increases [78]. Liu et al. demonstrated that CeO_2_ nanoparticles stabilised with curcumin and PLGA also scavenge alkyl peroxyl radicals [79]. Kang et al. found that glycol-capped cerium dioxide nanoparticles reduce the concentration of intracellular reactive oxygen species in U937 cells and reactive nitrogen species in RAW 264.7 cells [80].

**Figure 6:**
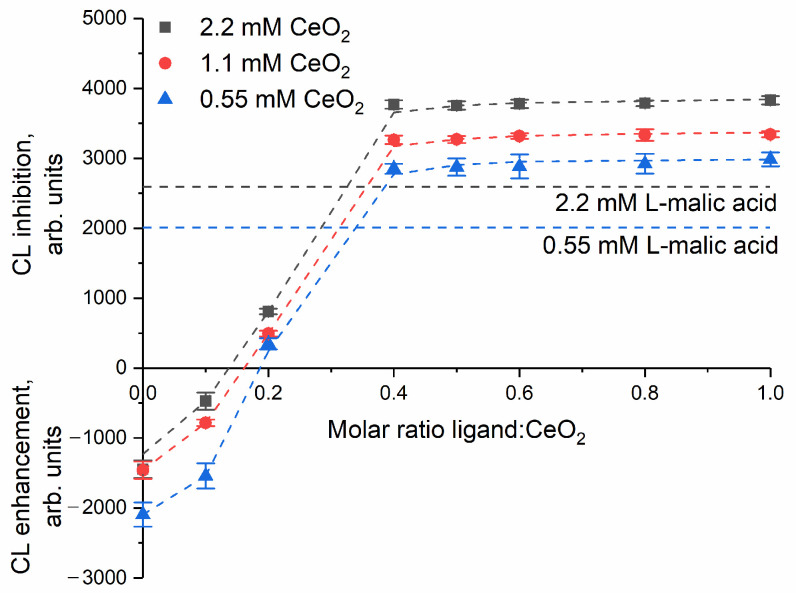
The chemiluminescence inhibition/enhancement for the malate-stabilised cerium dioxide sols at various ligand:CeO_2_ molar ratios. The chemiluminescence inhibition levels for the aqueous solutions of the bare malic acid are given for the reference.

A quantitative comparative analysis of the antioxidant activity of cerium dioxide sols was carried out by determining the values of antioxidant or pro-oxidant activity (capacity), which are calculated by integrating the inhibition area in the chemiluminescence curves over time. As can be seen from Figure 6, the antioxidant capacity increased with increasing ligand:CeO_2_ molar ratio and reached an almost constant level when the ligand:CeO_2_ ratio was 0.4:1. From Figure 6, it follows that bare CeO_2_ sol as well as CeO_2_ sol stabilised by malic acid at ligand:CeO_2_ ratio 0.1:1 exhibit pro-oxidant properties, while at higher ligand concentrations, at ligand:CeO_2_ ratios 0.2:1 and higher, the sols act as an antioxidant. This result is consistent with the complete coating of CeO_2_ particles with ligands at a molar ratio of 0.18:1 (see Table 1). Thus, the antioxidant activity of cerium dioxide sol increases with increasing the content of L-malic acid on ceria nanoparticles. Similarly, Kwon et al. showed that, with an increase in the ligand:CeO_2_ weight ratio from 0.5:1 to 3:1, the hydrodynamic diameter of triphenylphosphonium-conjugated ceria nanoparticles decreases [81]. The ceria nanoparticles stabilised with a weight ratio of 3:1 were localised in mitochondria, while nanoparticles stabilised with weight ratios of 1:1 and 0.5:1 were localised in lysosomes.

From Figure 6, it follows that the antioxidant capacity of the malate-stabilised ceria sols in the concentration range of 0.55–2.2 mM is approximately 1.5 times higher than that of the aqueous solutions of pure L-malic acid with the same concentration. This observation indicates that nanocrystalline ceria enhances the radical-scavenging ability of malic acid.

**Figure 7:**
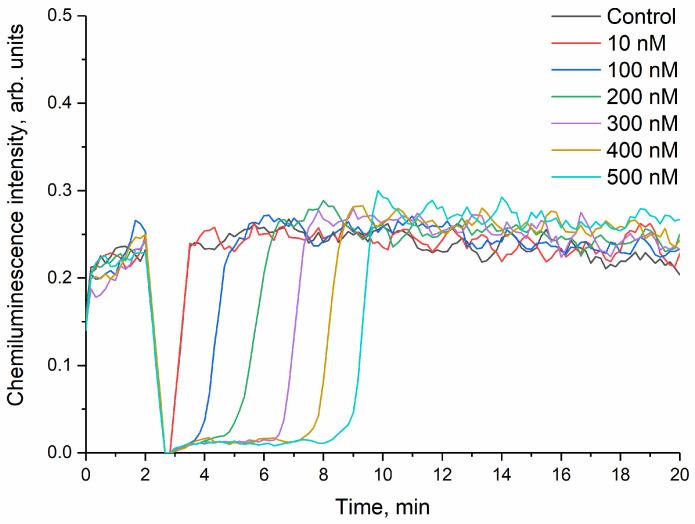
Kinetics of the luminol oxidation product chemiluminescence in a reaction mixture containing a Tris-HCl buffer solution (pH 7.4), ABAP and sodium ascorbate. The chemiluminescence signal was recorded at 36 °C. As the control, the solution containing Tris-HCl, ABAP and luminol was used, without the addition of sodium ascorbate.

Water-soluble sodium ascorbate was used to determine the antioxidant capacity of malate-stabilised cerium dioxide in concentration units of this standard antioxidant [82]. When sodium ascorbate solution was added to the reaction mixture containing a Tris-HCl solution, ABAP and luminol, a decrease in chemiluminescence intensity to almost zero was initially observed, after which the intensity increased to the chemiluminescence intensity of the control solution (Figure 7). The area of chemiluminescence inhibition was linearly dependent on the concentration of sodium ascorbate (Figure S3).

It is worth noting that the shape of chemiluminescence kinetic curves of luminol in the presence of a CeO_2_ sol stabilised by L-malic acid at a ligand:CeO_2_ molar ratio of 0.2:1 (Figure 5) coincides with the appearance of chemiluminescence kinetic curves of luminol in the presence of sodium ascorbate (Figure 7). Therefore, the antioxidant activity of cerium dioxide can be expressed in units of standard antioxidant concentration. The antioxidant capacity of 1 mM malate-stabilised cerium dioxide sol is equivalent to the antioxidant capacity of 0.012 ± 0.001 mM sodium ascorbate. Thus, the radical-scavenging activity of malate-stabilised CeO_2_ is 80 times lower than the antioxidant activity of sodium ascorbate.

**Figure 8:**
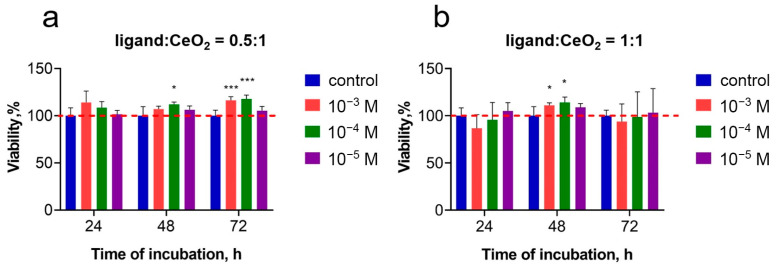
Metabolic activity assay of human MSCs after cultivation with malate-stabilised ceria nanoparticles (10^−3^–10^−5^ M) at various ligand-to-metal ratios (ligand:CeO_2_ = 0.5:1 (**a**); ligand:CeO_2_ = 1:1 (**b**)) after 24, 48 and 72 h, according to the MTT assay. * *p* ≤ 0.05, *** *p* ≤ 0.005.

A comprehensive cytotoxicity assay of malate-stabilised cerium dioxide sols was performed on human mesenchymal stem cells (MSCs) after 24, 48 and 72 h of cultivation (Figure 8). The analysis of the metabolic activity of MSCs in the presence of malate-stabilised ceria (10^−3^−10^−5^ M) nanoparticles indicated a high level of cell viability at all of the ligand:CeO_2_ ratios tested. Even at high concentrations of ceria nanoparticles (10^−3^ M), no significant decrease in the cells’ metabolic activity and viability was observed. Moreover, at all of the concentrations tested, the nanoparticles showed a weak dose-dependent stimulating effect on the cells, which is mostly apparent after 48 h of cultivation. Such a delayed stimulation is presumably due to the relatively long cell cycle inherent in MSCs [83,84]. The authors have previously shown that citrate-stabilised cerium oxide nanoparticles are capable of stimulating the proliferation of human MSCs and mouse embryonic fibroblasts, providing a 2-fold increase in their proliferative activity [85,86]. The molecular mechanisms underlying this effect are associated with the antioxidant properties of CeO_2_ nanoparticles and their ability to reduce elevated levels of intracellular ROS, which are formed when cells are isolated into culture and persist during their cultivation in vitro, as well as during their reseeding and other technical manipulations. Reducing excess levels of intracellular ROS in the presence of CeO_2_ nanoparticles provides conditions that are as close as possible to the native state of cells in vivo, which promotes their active proliferation.

**Figure 9:**
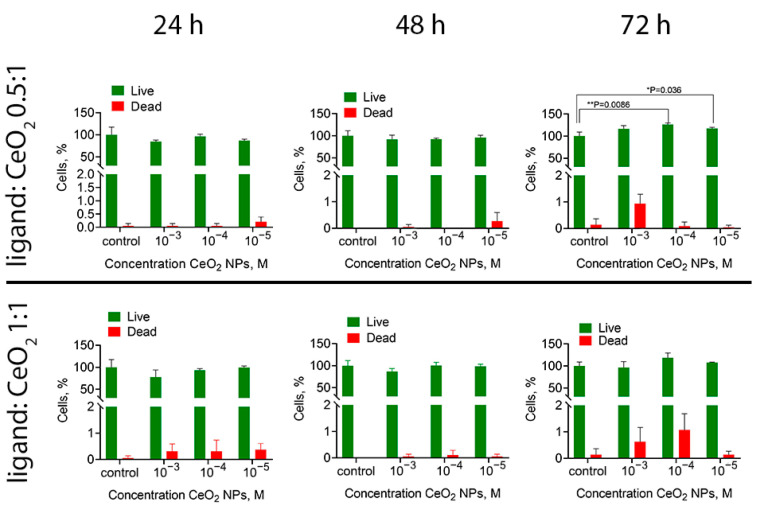
Live/Dead assay for the human MSCs after 24, 48 and 72 h of cultivation with different concentrations of malate-stabilised CeO_2_ nanoparticles with various ligand-to-metal ratios.

A viability analysis (Live/Dead assay) was performed on human MSCs after their cultivation with malate-stabilised ceria nanoparticles at various concentrations of the nanoparticles (10^−3^–10^−5^ M) and ligand:CeO_2_ molar ratios (0.5:1, 1:1). A quantitative assessment of the proportion of living and dead cells is shown in Figure 9 (for the microscopy images of the cells stained with Hoechst 33342 or propidium iodide, refer to Figure S4 in the Supplementary Materials). After 72 h of cultivation of the cells with malate-stabilised ceria nanoparticles, a tendency was observed to the increase in the number of live cells, which supports the above results on the increased metabolic activity of the cells in the presence of the nanoparticles (see Figure 8).

The analysis of the morphological features of human MSCs after cultivation with malate-stabilised ceria nanoparticles did not reveal pathological changes for any CeO_2_ concentrations tested (10^−3^–10^−5^ M): the cells retained their spindle shape and normal nuclear–cytoplasmic ratio (Figure S4). At the same time, comparing the data of the Live/Dead assay (Figure 9) with the results of the MTT assay (Figure 8) suggests that malate-stabilised ceria nanoparticles had a fairly high degree of biocompatibility.

The data from chemiluminescent analysis of the antioxidant activity of CeO_2_ sols towards alkyl peroxyl radicals revealed the possibility of quantitative regulation of radical-scavenging properties by changing the ligand-to-metal ratio for L-malic acid and ceria nanoparticles. The results of the analysis of the biocompatibility of malate-stabilised cerium dioxide sols demonstrate that ceria sols stabilised with L-malic acid at ligand:CeO_2_ molar ratios of 0.5:1 and 1:1 do not cause a significant decrease in the metabolic activity and viability of human mesenchymal stem cells during 24, 48 and 72 h of cultivation. This confirms their high level of biocompatibility. At the same time, malate-stabilised CeO_2_ sols stimulate human mesenchymal stem cell proliferation, which suggests the possibility of using this type of nanomaterial in various biomedical applications, including cellular technologies and regenerative medicine.

## 4. Conclusions

In this study, a quantitative assessment of the antioxidant activity of malate-stabilised cerium dioxide sols was carried out for the first time using the chemiluminescent method. It has been shown that the radical-scavenging properties of colloidal solutions of cerium dioxide stabilised with L-malic acid (ligand:CeO_2_ molar ratios of 0.2:1, 0.4:1, 0.5:1, 0.6:1, 0.8:1 and 1:1) are determined by the ligand-to-metal ratio of L-malic acid and ceria nanoparticles. The radical-scavenging properties of malate-stabilised cerium dioxide obtained at a ligand:CeO_2_ molar ratio of 0.2:1 are two orders of magnitude lower than the antioxidant activity of sodium ascorbate. Cerium dioxide nanoparticles stabilised with L-malic acid do not demonstrate cytotoxic effects against human mesenchymal stem cells, in a wide range of concentrations (10^−3^−10^−5^ M), and they are able to stimulate their proliferation after 72 h of cultivation.

The results presented here demonstrate that a mere alteration in the CeO_2_-to-ligand molar ratio can be employed to switch the redox properties of the nanoparticles, thereby facilitating the development of novel biologically active nanomaterials. The results are also of particular interest in light of the ongoing debate surrounding the enzyme-like (and, more specifically, oxidoreductase-like) behaviour of nanomaterials.

## Figures and Tables

**Table 1:** Estimation of the ligand:CeO_2_ molar ratio required to ensure the complete coating of CeO_2_ nanoparticles with a diameter of 2.8 nm.

D_XRD_, Å	a, Å	TPSA *, Å^2^	ligand:CeO_2_
28	5.441	94.8	0.18:1

* Topological polar surface area (TPSA) for L-malic acid is 94.8 Å^2^ [66].

## Data Availability

The raw experimental data are available upon request to the corresponding author.

## Supplementary Materials

The following supporting information can be downloaded at: https://www.mdpi.com/article/10.3390/nano14231908/s1, Table S1: ζ-potential of the nanoparticles at various ligand:CeO_2_ molar ratios; Figure S1: STEM images of CeO_2_ powders obtained by drying ceria sols stabilised with L-malic acid at molar ratios ligand:CeO_2_: 0:1 (a), 0.2:1 (b), 0.4:1 (c) and 1:1 (d); Figure S2: FTIR spectra of malate-stabilised ceria samples (molar ligand:CeO_2_ ratios are provided), L-malic acid and monosubstituted ammonium malate. Figure S3: Dependence of the antioxidant activity of sodium ascorbate on its concentration; Figure S4: Live/Dead assay for the human MSCs after 24, 48 and 72 h of cultivation with different concentrations of malate-stabilised CeO_2_ nanoparticles with various ligand-to-metal ratios.
