# Supplementary material for: Ligand-to-Metal Ratio Governs Radical-Scavenging Ability of Malate-Stabilised Ceria Nanoparticles
Source: Nanomaterials (Basel). 2024 Nov 27;14(23):1908. doi: 10.3390/nano14231908 (PMC11643014; doi:10.3390/nano14231908)
Supplement: Supplementary file 1 [file nanomaterials-14-01908-s001.zip › nanomaterials-3251019-supplementary.pdf]

Supplementary Information

Ligand-to-Metal Ratio Governs Radical-Scavenging Ability of Malate-Stabilised Ceria Nanoparticles

A.D. Filippova [1], A.E. Baranchikov [1,*], M.A. Teplonogova [1], I.V. Savintseva [2], A.L. Popov [2] and V.K. Ivanov [1]

[1] Kurnakov Institute of General and Inorganic Chemistry, Russian Academy of Sciences, Leninsky Prospect, 31, Moscow, 119991, Russia
[2] Institute of Theoretical and Experimental Biophysics, Russian Academy of Sciences, Institutskaya str., 3, Pushchino, 142290, Russia

Table S1. ζ-potential of the nanoparticles at various ligand:CeO_2 molar ratios.

**ligand:CeO_2**	**pH**	**ζ-potential, mV**
1:1	7.4	−16±1
0.8:1	7.4	−17±1
0.6:1	7.5	−16±1
0.5:1	7.4	−17±1
0.4:1	7.5	−17±1
0.2:1	7.5	−13±1
0.1:1	7.6	–7±2
0:1	2.0	+18±1

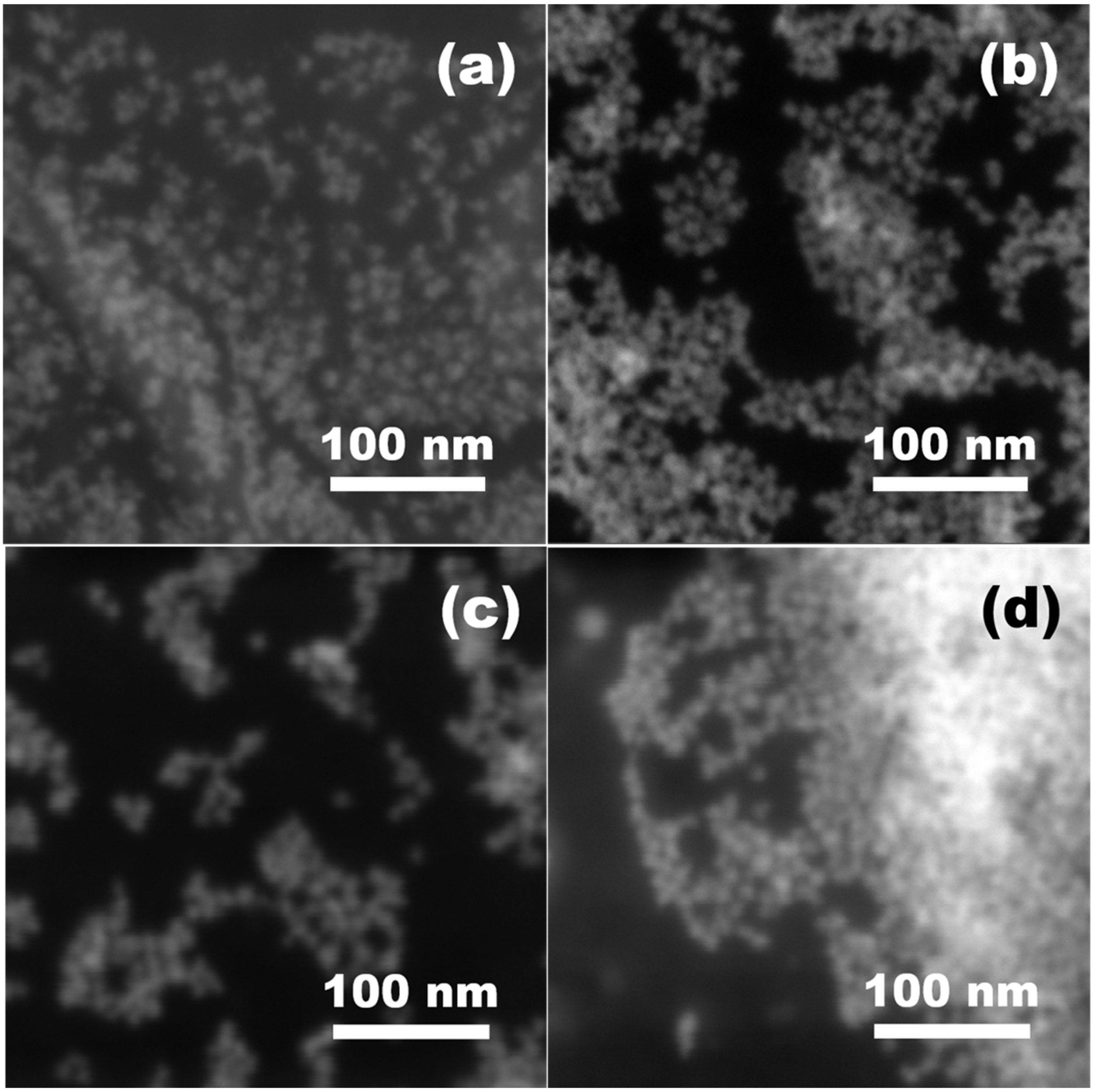

Figure S1. STEM images of CeO_2 powders obtained by drying ceria sols stabilised with L-malic acid at molar ratios ligand:CeO_2: 0:1 (**a**), 0.2:1 (**b**), 0.4:1 (**c**) and 1:1 (**d**).

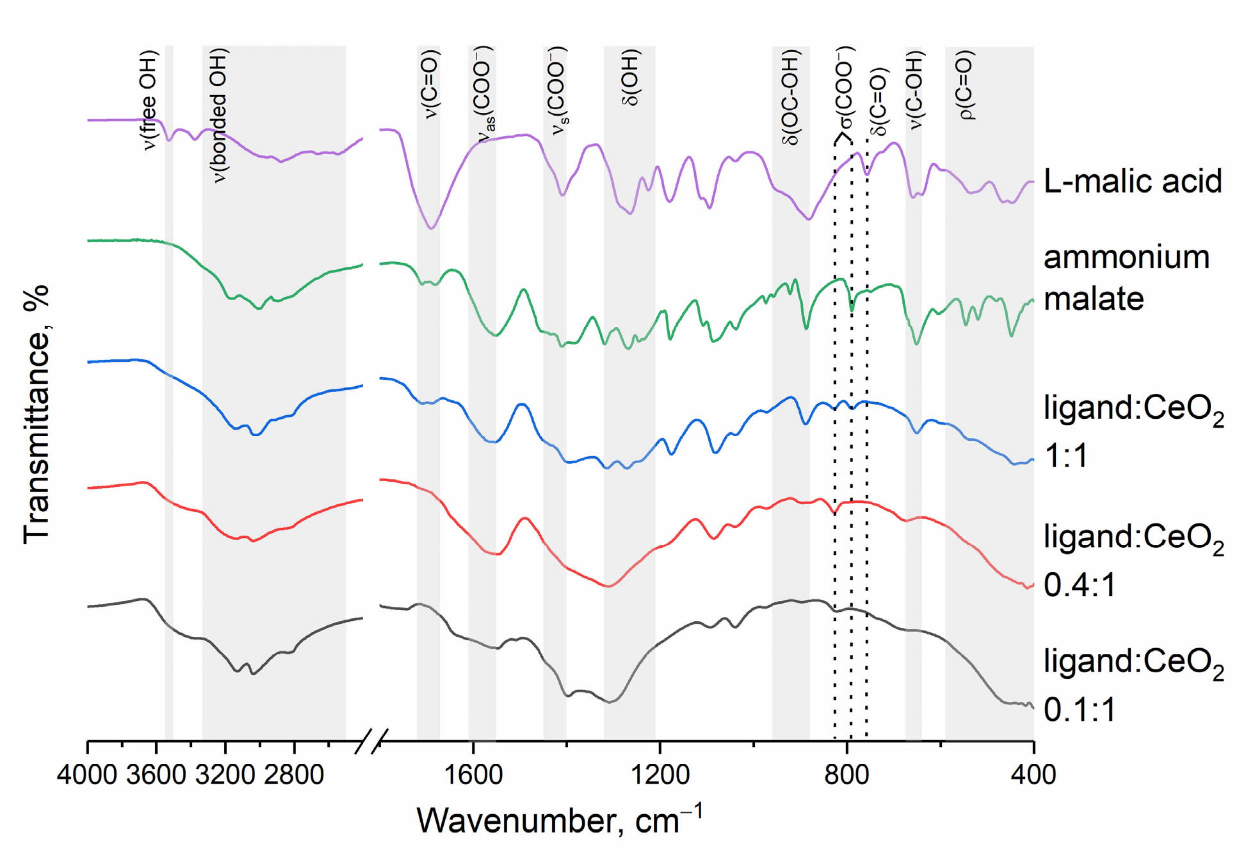

Figure S2. FTIR spectra of malate-stabilised ceria samples (molar ligand:CeO_2 ratios are provided), L-malic acid and monosubstituted ammonium malate.

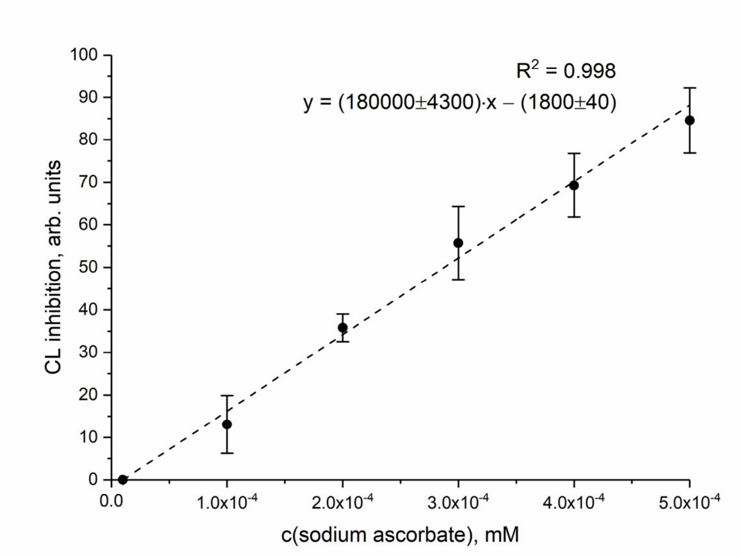

Figure S3. Dependence of the antioxidant activity of sodium ascorbate on its concentration.

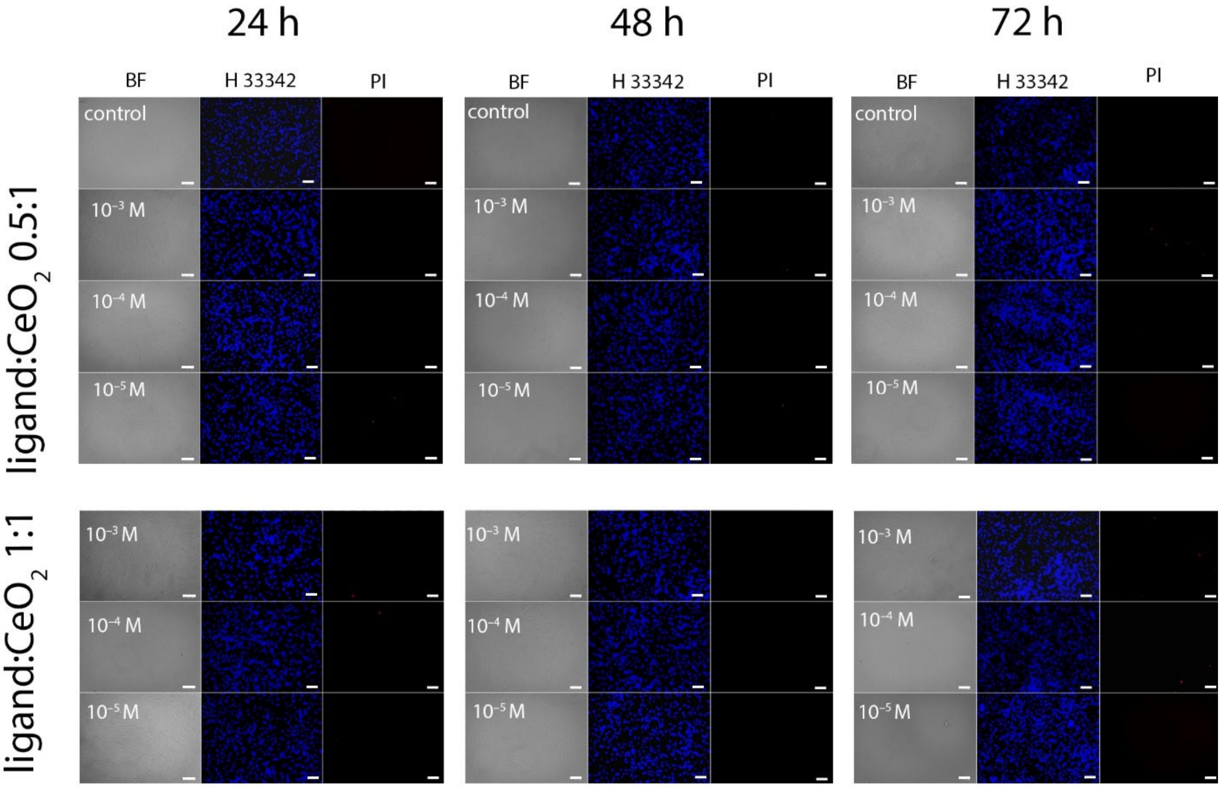

Figure S4. Live/Dead assay for the human MSCs after 24, 48 and 72 h of cultivation with different concentrations of malate-stabilised CeO_2 nanoparticles with various ligand-to-metal ratios.
